# Urokinase-type plasminogen activator (uPA) regulates invasion and matrix remodelling in colorectal cancer

**DOI:** 10.1016/j.mbplus.2023.100137

**Published:** 2023-11-15

**Authors:** Auxtine Micalet, Luke J. Tappouni, Katarzyna Peszko, Despoina Karagianni, Ashley Lam, John R. Counsell, Sergio A. Quezada, Emad Moeendarbary, Umber Cheema

**Affiliations:** a UCL Centre for 3D Models of Health and Disease, Department of Targeted Intervention, Division of Surgery and Interventional Science, University College London, Charles Bell House, 43-45 Foley Street, London W1W 7TS, United Kingdom; b Department of Mechanical Engineering, University College London, Gower Street, London WC1E 6BT, United Kingdom; c UCL Centre for Targeted Cancer Therapies, Department of Targeted Intervention, Division of Surgery and Interventional Science, University College London, Charles Bell House, 43-45 Foley Street, London W1W 7TS, United Kingdom; d Immune Regulation and Tumour Immunotherapy Group, UCL Cancer Institute, University College London, 72 Huntley Street, London WC1E 6DD, United Kingdom; e 199 Biotechnologies Ltd., Gloucester Road, London W2 6LD, United Kingdom

**Keywords:** 3D models, Tumour microenvironment, CRISPR-Cas9, Urokinase-type plasminogen activator, uPA, Cancer invasion, Mechano-based cancer therapy, Stiffness

## Abstract

**Background:**

Cancer cells remodel their local physical environment through processes of matrix reorganisation, deposition, stiffening and degradation. Urokinase-type plasminogen activator (uPA), which is encoded by the *PLAU* gene, is an extracellular proteolytic enzyme known to be involved in cancer progression and tumour microenvironment (TME) remodelling. Perturbing uPA therefore has a strong potential as a mechano-based cancer therapy. This work is a bioengineering investigation to validate whether 1) uPA is involved in matrix degradation and 2) preventing matrix degradation by targeting uPA can reduce cancer cell invasion and metastasis.

**Methods:**

To this aim, we used an engineered 3D *in vitro* model, termed the tumouroid, that appropriately mimics the tumour’s native biophysical environment (3 kPa). A CRISPR-Cas9 mediated uPA knockout was performed to introduce a loss of function mutation in the gene coding sequence. Subsequently, to validate the translational potential of blocking uPA action, we tested a pharmacological inhibitor, UK-371,801. The changes in matrix stiffness were measured by atomic force microscopy (AFM). Invasion was quantified using images of the tumouroid, obtained after 21 days of culture.

**Results:**

We showed that uPA is highly expressed in invasive breast and colorectal cancers, and these invasive cancer cells locally degrade their TME. *PLAU* (uPA) gene knock-out (KO) completely stopped matrix remodelling and significantly reduced cancer invasion. Many invasive cancer gene markers were also downregulated in the *PLAU* KO tumouroids. Pharmacological inhibition of uPA showed similarly promising results, where matrix degradation was reduced and so was the cancer invasion.

**Conclusion:**

This work supports the role of uPA in matrix degradation. It demonstrates that the invasion of cancer cells was significantly reduced when enzymatic breakdown of the TME matrix was prevented. Collectively, this provides strong evidence of the effectiveness of targeting uPA as a mechano-based cancer therapy.

## Highlights

• Invasive breast and colorectal cancer cells locally degrade their tumour microenvironment.
• Urokinase-type plasminogen activator (uPA) is overexpressed in invasive cancer cells.
• Targeting uPA stops matrix degradation.
• Targeting uPA limits cancer invasion.
• uPA therefore has high potential as a target for mechano-based cancer therapies.

## Introduction

As a tumour forms and grows, its tumour microenvironment (TME) is remodelled leading to an increased local stiffness[1], [2], [3], [4]. Extracellular matrix (ECM) deposition, ECM cross-linking, and force-mediated physical remodelling[4] all participate in the stiffening. This strong desmoplastic reaction at the tumour border creates a dense fibrotic ‘shield’ that limits the access of immune cells to the tumour, impacts chemical signalling, and reduces therapy delivery and efficacy. The increased stiffness triggers epithelial-to-mesenchymal transition (EMT) in epithelial cancer cells, allowing them to acquire mesenchymal characteristics with aberrated cell contractility and motility. Increased cellular contractility allows cells to break free from the primary tumour as a single cell and motility enables them to invade out[1], [3], [5], [6]. At this point however, the ECM architecture can physically restrict invasion due to its stiff and dense stroma associated with reduced pore size. Therefore, local proteolytic degradation is required for opening-up passages in the matrix, allowing cell migration [4], [5]. Previous research reports that, when introduced in a physiologically relevant 3D matrix, less invasive HT-29 cancer cells stiffen their TME by 60 % whilst highly invasive HCT 116 cancer cells soften the matrix by over 40 % after 21 days of culture [5].

**Fig. 1:**
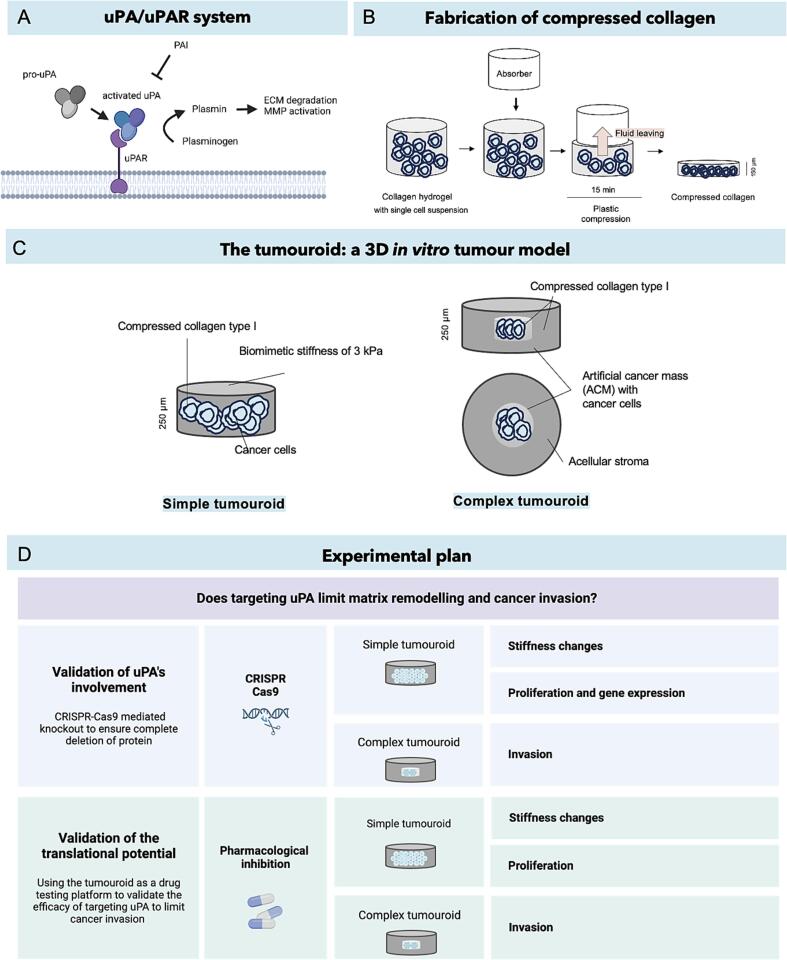
**Schematics of concepts and experimental plan**. **A)** Schematic of the uPA/uPAR system. uPA is a protein linked to ECM degradation and cancer invasion. **B)** Schematic of the fabrication process to obtain a compressed, dense collagen matrix. The gels are in the first instance set as collagen hydrogels (2 mg/mL of collagen type I), with cells embedded within them. A RAFT^TM^ absorber is then placed on the gel for 15 min, during which time the gel is compressed via fluid being absorbed out. The resulting gel is a ∼150 μm gel of dense collagen matrix, previously reported to be ∼3 kPa (vs ∼200 Pa for the hydrogel). **C)** Schematic of the 3D model used for this work, and its two iterations: the simple tumouroid and the complex compartmentalised tumouroid. The matrix used is compressed collagen type I, which is biomimetic in terms of collagen density and stiffness. A complex tumouroid is made of a central artificial cancer mass (ACM) in which the cancer cells are embedded. Over time, the cells start invading out of the cancer mass and into the stroma, which represents healthy tissue. This invasion can be monitored and quantified through imaging. **D)** Schematic of the experimental plan. *PLAU* (uPA) was knocked-out using CRISPR-Cas9 as well as inhibited via a pharmacological drug to explore the translational value of targeting uPA. Changes in stiffness and invasion were measured for both approaches.

The unique remodelling processes of local TME stiffening and ECM degradation are attributed to proteins such as transforming growth factor beta (TGF-β) [7], lysyl oxidase (LOX) [8], [9], [10], [11], [12], metalloproteinase inhibitors (TIMPs) [13] and matrix metalloproteinases (MMPs) [5], [14], [15]. Urokinase-type plasminogen activator (uPA) is an extracellular proteolytic enzyme also known to be involved in the TME remodelling and in cancer progression [16], [17], [18], [19], [20]. uPA is part of the plasminogen activation system, also called the uPA/uPAR system (Fig. 1A). Pro-uPA binds to the cell surface receptor uPAR and transforms into an activated urokinase-type plasminogen activator (uPA). uPA in turn converts plasminogen to plasmin, which is a protein with many functions, including degradation of fibrin and activation of MMPs by transforming pro-MMPs into active MMPs [16], [17], [18], [19], [20]. Natural inhibitors of uPA are plasminogen activator inhibitors PAI-1 and PAI-2.

Disrupting the cells' capacity to remodel the matrix or specifically targeting the stiffness of the ECM represents a promising approach in the emerging field of mechano-based therapies [21]. Matrix metalloproteinase, as the main matrix cleaving enzyme family, is a high-potential therapeutic target with over 50 MMP inhibitors currently being investigated [22]. The role of MMPs in matrix remodelling in cancer has been studied in depth in our model, notably by using a broad-spectrum inhibitor BB-94 [5]. Urokinase-type plasminogen activator (uPA) is a less studied target, with only a handful of commercially available inhibitors. Masucci *et al.* in 2022 reported a total of four uPA/uPAR system inhibitors currently in clinical trial: WX-UK1, WX-671, Å6 and 68 Ga-NOTA- AE105 [19].

The aim herein was to target uPA to validate its involvement in the cancer cells’ ability to locally degrade their TME and correlate that to their ability to migrate out of the primary tumour and metastasise. uPA could then be considered an impactful mechano-based therapy candidate. To the best of our knowledge, we are the first to report on the impact of uPA on tumour micro-environment stiffness, by directly measuring matrix stiffness when uPA is present versus when uPA is inhibited or knocked-out.

To address this aim, we used an engineered 3D *in vitro* model that more appropriately mimics the tumour’s native biophysical environment (Fig. 1B and C). Using a 3D model of relevant matrix architecture and stiffness is particularly important when studying the effects of matrix remodelling. Our 3D model, called the tumouroid, is highly biomimetic. Its main ECM composition is monomeric collagen type I, and its ECM fibre density and matrix stiffness replicates *in vivo* tumour tissue structural and mechanical properties [5]. The stiffness of malignant epithelial tissue is indeed reported to be 1 to 10 kPa [23], [24], [25], [26], [27] which is similar to the stiffness of our model that is 3 kPa^5^. As a point of comparison, Matrigel, the gold standard matrix for organoids, is 40 Pa^5^, ∼100 fold softer than tumour tissue. The dense collagen environment in our tumoroid model is obtained through plastic compression of collagen I hydrogel, using RAFT^TM^ absorbers (Fig. 1B). uPA’s role in cancer matrix remodelling was validated using CRISPR-Cas9 to delete the gene and ensure no protein expression. A HCT 116 monoclonal knockout cell line was generated in-house to this effect. A pharmacological drug approach was subsequently employed to determine the translational potential of targeting uPA. For both approaches, the tumouroids were embedded with cancer cells (i.e. WT or KO // with or without drug treatment) and cultured for 21 days before characterising invasion and matrix remodelling. To measure the changes in matrix stiffness, atomic force microscopy (AFM) was used. Invasion was quantified from imaging of the tumouroid after 21 days of culture. This is illustrated in Fig. 1D.

## Results

### Matrix remodelling and uPA expression is high in invasive epithelial cancer cells

**Fig. 2:**
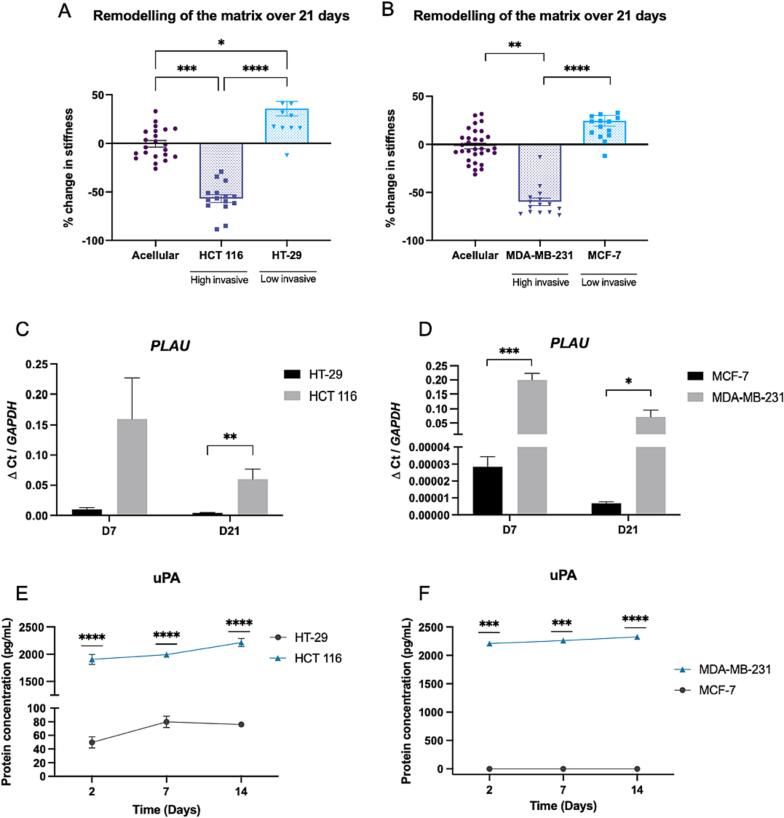
**Matrix remodelling and uPA expression in epithelial cancer cells**. **A-B)** Percentage change in stiffness of A. colorectal cancer and B. breast cancer tumouroids, after 21 days of culture, measured by AFM (n = 3 biological replicates, 16 measurements per sample, Kruskal-Wallis significance). Percentage change calculated from acellular controls. **C-D)** Relative gene expression (ΔCt, normalised to *GAPDH*) of *PLAU* (uPA) at day 7 and day 21 in colorectal and breast cancer tumouroids (n = 3 biological replicates, n = 3 technical replicates, unpaired *t* test significance). **E-F)** uPA protein expression over 14 days in colorectal and breast tumouroids (n = 3 biological replicates, technical duplicates, unpaired *t* test (colorectal) and one sample *t* test (breast) significance). All p-value significance is indicated as: * <0.05, ** <0.01, *** <0.001, **** <0.0001.

Simple breast and colorectal epithelial cancer tumouroid models were engineered with either less invasive cancer cells (HT-29 and MCF-7) or more invasive cancer cells (HCT 116 and MDA-MB-231; difference in invasiveness shown in Fig. S1). They were cultured for 21 days, during which the cancer cells remodelled their extracellular matrix. At day 21 the tumouroids, alongside acellular controls (all n = 3), were measured by atomic force microscopy (AFM). It was observed that the less invasive cancer cells HT-29 stiffened their TME (36 % stiffening compared to the acellular control, p = 0.0201, Fig. 2A). The less invasive MCF-7 cells also showed a trend of stiffening of the matrix, although this was not significant (24 % stiffening compared to the acellular control, Fig. 2B). The highly invasive cancer cells however significantly degraded their matrix (-59 % softening compared to the acellular control for MDA-MB-231 cells, p = 0.0012, and −57 % for HCT 116 cells, p = 0.0001 Fig. 2A and B). This remodelling pattern of matrix stiffening for less invasive cells and matrix degradation for highly invasive cancer cells is in line with previously published data [5]. Proliferation rate does not impact the observed matrix stiffness, as previously demonstrated [5].

Urokinase-type plasminogen activator, encoded by the gene *PLAU,* is a matrix degradation enzyme that is highly expressed in the invasive cancer cells during their 21 days in the tumouroid. At the gene level, *PLAU* was upregulated at day 7 and day 21 in both highly invasive cells, HCT 116 and MDA-MB-231, compared to their corresponding less invasive cells (significant upregulation observed at day 21 for HCT 116, p = 0.0282, and MDA-MB-231 at day 7, p = 0.0010 and day21, p = 0.0412, Fig. 2C and D). At the protein level, an ELISA assay using media collected from the tumouroids at days 2, 7 and 14 showed significantly higher protein concentration at all timepoints for the highly invasive cells HCT 116 and MDA-MB-231 (Fig. 2E and F).

The hypothesis herein is that for early onset cancer, the 3D micro-environment is stiffened, creating a shield that protects the cancer cells against external factors [1], [4], [5], [28]. However, as the cancer cells become more invasive and migrate out of the primary tumour, the dense, stiff microenvironment physically restricts invasion. The cancer cells therefore start locally degrading their ECM to open up passages for cell migration [4], [5]. Urokinase-type plasminogen activator is a degradation enzyme that is highly expressed in invasive cancer cells, suggesting a role in the observed ECM degradation. Therefore, targeting uPA could both stop matrix remodelling and limit cancer invasion.

The study here onwards focused on colorectal cancer cells only, particularly the invasive HCT 116 cells. However, this work is likely transferable to other epithelial cancer types as uPA is also highly expressed in the invasive epithelial breast cancer model.

### Generating a monoclonal uPA (*PLAU*) HCT 116 knockout cell line using CRISPR-Cas9

To study the role of uPA in matrix remodelling and cancer cell invasion, its corresponding gene *PLAU* was knocked out in a HCT 116 cell line by CRISPR-Cas9. As shown above, HCT 116 are highly invasive epithelial cancer cells, expressing high mRNA and protein levels of uPA. CRISPR-Cas9 induces double-stranded breaks (DSBs), which results in the formation of insertions and deletions (indels). In some cases, a frameshift mutation occurs that disrupts the gene sequence and leads to a non-functional protein. Note that HCT 116 cells are near diploid cells [29].

**Fig. 3:**
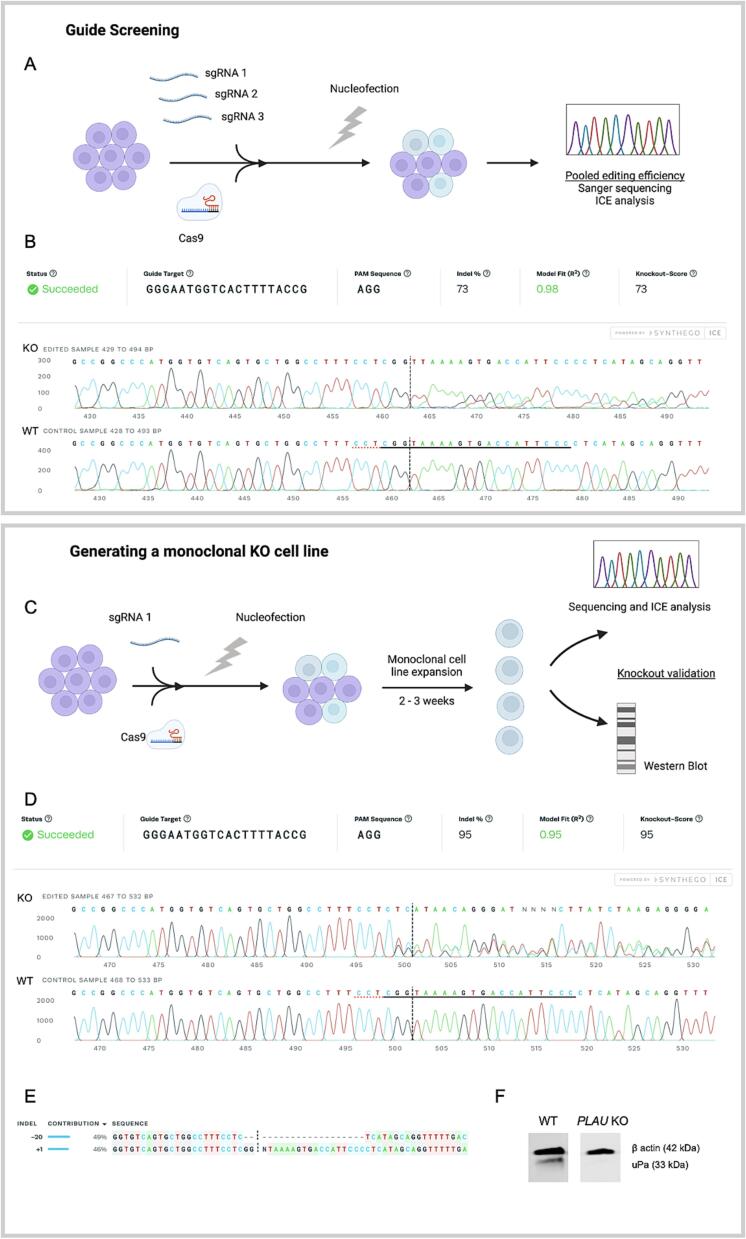
**Knockout of uPA (*PLAU*) in HCT 116 cells using CRISPR-Cas9. A)** Schematic of the methodology used to screen guide RNAs. **B)** ICE analysis demonstrating the efficiency of the most efficient guide sequence, AC. The horizontal black underlined region represents the guide sequence, preceded by the PAM sequence underlined with a red dotted line. The vertical black dotted line represents the cut site. **C)** Schematic of the methodology used to generate a monoclonal (MC) knockout cell line. **D)** ICE analysis of the selected monoclonal cell line vs wild type. **E)** Breakdown of indel distribution in the chosen clone, with a −20 deletion/+1 insertion, taken from ICE analysis. **F)** Western blot showing loss of uPA protein expression in the KO cell line. (For interpretation of the references to colour in this figure legend, the reader is referred to the web version of this article.)

Three CRISPR guide RNAs (gRNAs) were tested, with their editing efficiency determined by Interference of CRISPR Edits (ICE) analysis (Fig. 3A and B, Fig. S2). A limiting dilution was performed on the population with the highest editing efficiency, AC at 73 % knock-out efficiency (Fig. 3C), to generate monoclonal populations [30]. Following the limiting dilution, seven clones were screened via a western blot to validate the loss of protein expression (Fig. S3). One of the clonal populations was identified as a knock-out due to its lack of uPA expression (Fig. 3F). This was further confirmed by the sequencing data (Fig. 3D). Indeed, a 20 bp deletion/1 bp insertion in *PLAU* was identified, giving this monoclonal population a 95 % knockout score (Fig. 3D and E).

### *PLAU* knock-out prevents matrix remodelling and significantly reduces cancer invasion

**Fig. 4:**
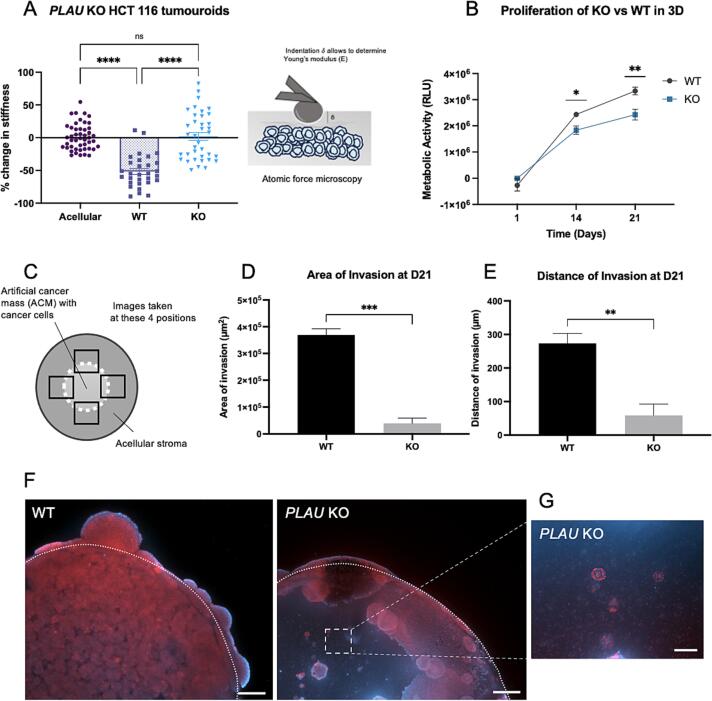
***PLAU* knockout stops matrix remodelling and significantly reduces cancer invasion A)** Percentage change in HCT 116 wild type (WT) and HCT 116 *PLAU* knockout (KO) tumouroids, after 21 days of culture, measured by AFM (n = 3 biological replicates, >16 measurements per sample, Kruskal-Wallis significance). Percentage change calculated from acellular controls. Schematic to the right: Schematic of atomic force microscopy. A bead (50 μm in diameter) glued at the end of a cantilever indents the surface of the sample. The indentation depth is then correlated back to stiffness (Young’s modulus, E) by fitting the Hertz model to the acquired force-curves. **B)** Proliferation of KO vs WT HCT 116 in 3D over 21 days, measured by CellTiter Glo (n = 3 biological replicates, technical duplicates, Mann-Whitney significance at each time point). **C)** Schematic of a complex, compartmentalised tumouroid (top view), with a central artificial cancer mass, where the cancer cells are initially segregated and a stroma into which the cells invade over 21 days. Images are taken at four positions, and at each position both maximum distance and total area of invasion is measured. An average is calculated per sample, and statistics are performed over 3 biological replicates. **D-E**) Area and distance of invasion of WT and *PLAU* KO cancer cells after 21 days (n = 3 biological replicates, 4 measurements per sample, showing unpaired *t*-test significance). **F)** Images of WT and *PLAU* KO tumouroids at day 21. The dotted line represents the initial ACM-stroma border. Reduced invasion is observed for the KO tumouroid. Red = Pan-cytokeratin, Blue = nuclei, scale bar = 200 μm. **G)** Image at the centre of a KO ACM. Showing that the cells are single cells rather than cells clustered in spheroids. Red = Pan-cytokeratin, Blue = nuclei, scale bar = 100 μm. All p-value significance is indicated as: * <0.05, ** <0.01, *** <0.001, **** <0.0001. (For interpretation of the references to colour in this figure legend, the reader is referred to the web version of this article.)

Simple tumouroids were set up with either wild type (WT) invasive HCT 116 cancer cells, or *PLAU* knockout (KO) HCT 116 cells, alongside acellular controls. After 21 days of culture, AFM was performed on the tumouroids to determine the change in matrix stiffness with or without uPA expression. The WT tumouroids were significantly softer than the acellular controls (-51 % softer, p < 0.0001, Fig. 4A). However, when *PLAU* was knocked out, matrix softening was entirely stopped (no change in matrix stiffness was observed compared to the acellular control, Fig. 4A). Viability of cells within the tumouroid by day 21 was confirmed via LIVE/DEAD staining (Fig. S4). Complex tumouroids were also engineered, with either WT or KO HCT 116 cells in the artificial cancer mass (ACM). The invasion of cancer cells from the artificial cancer mass into the stroma was observed and quantified after 21 days (Fig. 4C – G). Images were taken at four positions per sample to randomise the observations. The four values obtained per sample were averaged and then significance was determined over the three biological replicates. Per image, the maximum distance of invasion from the tumour border as well as the overall area of invasion was measured. This is illustrated Fig. 4C. *PLAU* deletion significantly reduced both the area and the distance of invasion (Fig. 4D – E). Quantification of the area of the invasion showed significant reduction with *PLAU* KO (from 369×10^3^ μm^2^ to 39×10^3^ μm^2^, p = 0.0004, Fig. 4D). The distance of invasion also decreased when *PLAU* was knocked-out (from 273 μm to 58 μm, p = 0.0088, Fig. 4E). This is demonstrated in Fig. 4F, which shows fluorescence staining of WT versus KO tumouroids at day 21. The initial ACM-stroma border is represented by the white dotted line. This reduced invasion is also shown in the mRNA levels of invasive markers. Trends were observed of KO cells having undergone less EMT than the WT cells in the tumouroid (shown in supplementary Fig. S5). They express more cytokeratin 20 (*KRT20*), which is an epithelial marker, and less vimentin (*VIM*) (Fig. S5B), which is an EMT marker, although not statistically significantly (p = 0.2232 and p = 0.1237 respectively). We also measured lower expression of matrix degradation and matrix cleaving enzyme *MMP9* in the KO cells compared to the WT, although again not statistically significantly (p = 0.4000).

It may seem like proliferation skews the invasion results, and although proliferation is slightly reduced in the KO cell line in 3D over 21 days (Fig. 4B), the reduced fluorescence in the centre of the KO ACM is due to the cells presenting as single cells rather than spheroids (Fig. 4G, close-up image at the centre of the ACM). Indeed, knocking out *PLAU* showed trends of reduced mRNA expression of cell-cell adhesion marker *EPCAM* (Fig. S5C, p = 0.3282).

This work confirms the role of uPA in matrix degradation, as with *PLAU* knocked out, the matrix was not softened. It also confirms the role of matrix degradation in cancer cell invasion, as when the cells could not locally break down the matrix and enlarge pore size, invasion was significantly reduced. Collectively, this points toward the effectiveness of targeting uPA as a mechano-based cancer therapy.

### Pharmacological inhibition of uPA as an effective mechano-based cancer therapy in a 3D *in vitro* model

The translation value of targeting uPA activity as an effective cancer therapy was tested through drug inhibition. UK-371,804, a small molecule uPA inhibitor with high potency and selectivity was used [31]. UK-371,804 specifically disrupts active uPA [31], as opposed to other drugs that competitively bind to the uPAR receptor, such as IPR-803 [32] or Angstrom6 A6 [30].

**Fig. 5:**
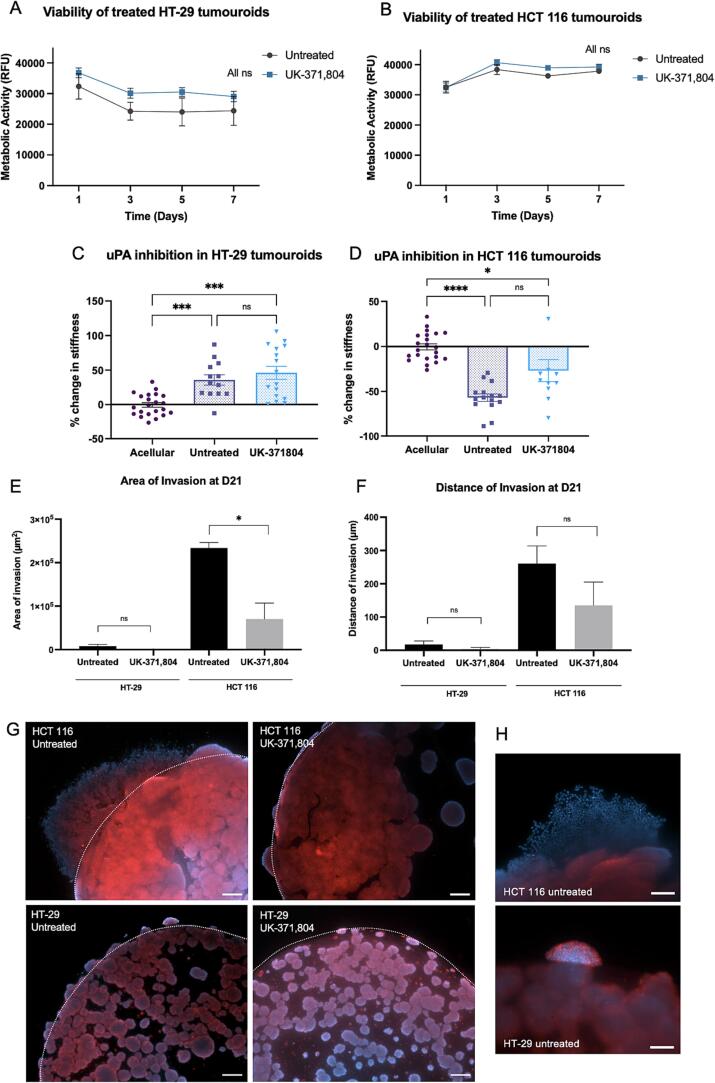
**Pharmacological inhibition of uPA as an effective mechano-based cancer therapy in a 3D tumouroid. A-B)** Viability of HT-29 and HCT 116 cells in a 3D tumouroid treated with 10 μM of UK-371,804, with no statistical differences between treated and untreated conditions (n = 3 biological replicates, technical duplicates, *t*-test at each time point) **C-D**) Percentage change in tumouroid stiffness after 21 days of culture with HT-29 or HCT 116 cells, either untreated or treated with 10 μM of UK-371,804. Measured by AFM (n = 3 biological replicates, ∼16 measurements per sample, Kruskal-Wallis significance). Percentage change calculated from acellular controls. **E-F)** Area and distance of invasion of cancer cells after 21 days with HT-29 or HCT 116 cells, either untreated or treated with 10 μM of UK-371,804 (n = 3 biological replicates, 4 measurements per sample, showing unpaired *t*-test significance). **G)** Images of HCT 116 and HT-29 tumouroids untreated vs treated with uPA inhibitor at day 21. The dotted line represents the initial ACM-stroma border. Reduced invasion is observed for the treated conditions. Red = Pan-cytokeratin, Blue = nuclei, scale bar = 200 μm. **H**) Close-up of the invasive phenotype of HCT 116 and HT-29 cells. HCT 116 are more invasive. Red = Pan-cytokeratin, Blue = nuclei, scale bar = 100 μm. All p-value significance is indicated as: * <0.05, *** <0.001, **** <0.0001. (For interpretation of the references to colour in this figure legend, the reader is referred to the web version of this article.)

The inhibitor concentration was optimized via a 2D cell viability assay (Fig. S6). The efficacy of UK-371,804 as a uPA inhibitor was proven using a uPA activity assay (Fig. S7). HCT 116 and HT-29 viability in 3D simple tumouroids treated with 10 μM UK-371,804 over 7 days was confirmed by a viability assay. No statistically significant reduction in metabolic activity was observed over these 7 days (Fig. 5A and B).

Simple tumouroids were constructed with HT-29 and HCT 116 cells and treated with 10 μM UK-371,804 every 48 h for 21 days. At day 21 the stiffness of these tumouroids, along with untreated controls and acellular controls, were measured by AFM. Untreated HT-29 stiffened the matrix (34 % stiffer than the acellular control, p = 0.0009, Fig. 5C), which echoes results from section 2.1 and Fig. 2. That remodelling pattern did not change when uPA was inhibited (n.s. between untreated and uPA inhibition, Fig. 5C). This was expected as HT-29 expressed only low levels of uPA and showed no matrix softening. HCT 116 untreated tumouroids soften over 21 days (-56 % for HCT 116 cells as compared to the acellular control, p = 0.0001, Fig. 5D). The UK-371,804 treatment resulted in less matrix degradation (-27 % softer than acellular controls, 29 % less softening compared to untreated control, Fig. 5D). Pharmacological inhibition of uPA did not lead to a complete elimination of matrix remodelling, as opposed to knocking out the gene, which was expected. Indeed, the drug binds to protein that is secreted rather than stops the expression of the protein. uPA protein will still be present in the supernatant, and potentially still be active depending on the temporality of the drug action. We can also note that the difference between untreated and treated is statistically non-significant, which is due to a large standard deviation. This is hypothesised to be partly due to non-homogenous diffusion of the drug within the sample, and partly due to the fact that uPA cannot be completely inhibited at all times.

Complex tumoroids were then used to investigate the effect of uPA inhibition on cancer cell invasion. The invasion of cancer cells from an artificial cancer mass (ACM) into acellular stroma was measured after 21 days. Minimal invasion was observed in control HT-29 tumoroids and the UK-371,804 treated HT-29 tumouroids (Fig. 5E and F), with no significant difference in area nor distance of invasion seen between both groups. In comparison, high invasion was observed in HCT 116 untreated tumouroids (Fig. 5E and F). uPA inhibition resulted in statistically significant reduction in the area of invasion of HCT 116 from 233.7 x10^3^ μm^2^ to 70.3 x10^3^ μm^2^ (p = 0.0136). The distance of invasion also shows a trend of decreased invasion when treated (from 260 μm to 134 μm). Fig. 5G shows fluorescence staining of the tumouroids, and the cellular invasion into the stroma. The initial ACM-stroma border is represented by the dotted white line. Fig. 5H shows a close up of the difference of invasion between the two cell lines. HCT 116 cells present a more invasive phenotype than HT-29 in terms of both distance and area, and a loss of cytokeratin, indicating EMT.

Through pharmacological inhibition of uPA, matrix remodelling was limited, and more importantly cancer cell invasion was reduced. This data suggests that uPA inhibition, specifically through drug action of UK-371,804, is effective at preventing cancer cells invasion from the primary tumour, and therefore reduces the risk of metastasis and formation of secondary tumours.

## Discussion and conclusion

A 2022 amendment to the FDA’s preclinical testing policy authorised drug tests performed in 3D *in vitro* models to go straight to human clinical trials, by-passing the need for animal testing [33]. The tumouroid model used herein is a highly biomimetic 3D model, that is easy to fabricate, manipulate and analyse. It allows for long term cultures (a month), and therefore long-term study of the effects of specific treatments. The tumouroid model provides a high-throughput testing platform for personalized healthcare, with the potential of testing various drugs, and drug concentrations on patient-specific cells. This work is a bioengineering investigation into the potential of urokinase-type plasminogen activator (uPA) protein as a cancer therapy target. The tumouroid model was employed to this effect.

Breast and colorectal tumouroids were engineered, with both less invasive epithelial cancer cells (HT-29 and MCF-7) and highly invasive epithelial cancer cells (HCT 116 and MDA-MB-231). Urokinase-type plasminogen activator, a matrix degradation enzyme and a cancer marker, was significantly upregulated in the more aggressive tumouroids, at both the gene and protein level. This was matched with stiffness measurements of the tumouroids, which showed a high degree of matrix softening which was linked to matrix degradation, by the highly invasive cancer cells. The less invasive cancer cells, however, stiffened their ECM. A hypothesis was made that matrix degradation was caused by uPA, and that matrix degradation was linked to cancer invasiveness.

To validate uPA’s involvement in matrix remodelling and cancer invasion, a CRISPR-Cas9 mediated uPA (gene name *PLAU*) knockout was performed in the highly invasive HCT 116 cancer cells, to ensure complete deletion of the gene and protein expression. Out of eight clones screened, only one monoclonal population was successfully validated as a complete KO (both at the gene and protein level). Ideally, the subsequent experiments should have been performed using more than one clone, to hedge against inter-clonal variability, notably in terms of phenotype. Two functional outputs were measured: changes in matrix stiffness by atomic force microscopy indentation tests, and changes in distance and area of invasion of cancer cells. It was observed that knocking out *PLAU* completely stopped matrix softening and significantly reduced cancer invasion. mRNA expression of invasive cancer markers such as vimentin, and other matrix cleaving proteins such as MMP9 was lower, although not statistically significant. uPA is therefore vital to the cancer cells for them to locally degrade their ECM, a characteristic that enables the cells to enlarge the pore size of the dense desmoplastic matrix, and create paths for them to migrate out.

As mentioned above, there is currently a strong push to validate potential targets using 3D models. Herein we subsequently aimed to target uPA via pharmacological inhibition. To this effect, we used the inhibitor UK-371,804. The drug concentration was validated in 2D and 3D and efficacy of the drug validated. Treatment of both less invasive and more invasive epithelial cancer tumouroids showed promising results. The UK-371,804 treated HCT 116 tumouroids displayed lower degree of matrix softening compared to the untreated tumouroids. This correlated with an observed reduction in cell invasiveness when uPA was inhibited. Compared to a gene knock-out, where the protein expression is entirely suppressed, a drug treatment is not as complete. Incomplete and inhomogeneous inhibition can be attributed to distribution and diffusion of the drug within the model. The temporal aspect of drug treatment also reduces the ability to block all protein, as proteins are secreted continuously by the cells whereas drug treatments are periodic. Future work will involve using the tumouroid model as a screening platform for uPA inhibition and will include using patient-specific cells and continuous distribution of the drug by culturing models within a bioreactor. We expect drug concentrations to be patient and disease stage specific.

Although this work was performed using colorectal cancer models, we hypothesise that uPA targeting will be similarly effective in other cancers, as uPA is overexpressed in breast cancer (section 2.1), prostate cancer [34], cervical cancer [35] and melanoma [36].

To conclude, this work demonstrates strong evidence that targeting urokinase-type plasminogen activator in cancer reduces matrix remodelling and limits invasiveness, and uPA is therefore a valid therapeutic target in cancer.

## Methods

*Cell Culture.* The HT-29 and HCT 116 human colorectal adenocarcinoma cell lines as well as the MDA-MB-231 human breast adenocarcinoma cell line were obtained from the ECACC (through Sigma Aldrich, Dorset, UK). The MCF-7 human breast adenocarcinoma cell line was gifted by Dr Nina Moderau and Mr Michael Toeller from Imperial College London. All cells were cultured in low glucose DMEM (1 g/L) supplemented with 10 % foetal bovine serum (FBS) and 1 % PenStrep (all reagents from Gibco^TM^ through Thermo Fisher Scientific, Loughborough, UK). Cells were cultured at 5 % carbon dioxide (CO_2_) atmospheric pressure and at 37 °C and passaged regularly in 2D monolayers. Cell-lines were routinely tested for mycoplasma infections.

*Engineering of Simple 3D Tumouroids.* Tumouroids were fabricated as previously described^5^. Briefly, 2 mg/mL collagen type I hydrogels were set up according to the RAFT^TM^ protocol (Lonza, Slough, UK). The collagen mix used to make these hydrogels was comprised of 80 % monomeric rat-tail collagen type-1 (First Link, Birmingham UK), 10 % 10X MEM (Sigma Aldrich, Dorset, UK), 6 % neutralizing solution, and 4 % cell suspension (at concentration of 270,000 cells per gel). Cells are indeed integrated as single cells, rather than pre-formed spheroids. Once polymerised for 15 min at 37 °C, the hydrogels were compressed for 15 min using RAFT^TM^ absorbers (Lonza, Slough, UK). The tumouroids were cultured for 21 days at 5 % CO_2_, 37 °C, with 50 % media changes every 48 h. Tumouroids were always set as biological triplicates.

*Engineering of Complex 3D Tumouroids.* Complex tumouroids were compartmentalised and used for invasion experiments. 96-well plate size compressed collagen gels (also called artificial cancer masses, or ACMs) were made with HCT 116 or HT-29 cells, at a concentration of 50,000 cells per gel. The ACMs were place in the centre of a 24-well plate sized hydrogel – the “stroma”, which is acellular. Once constructed and polymerised, the complex tumouroids were compressed using a RAFT^TM^ absorber. The tumouroids were cultured for 21 days at 5 % CO_2_, 37 °C. 50 % media changes were performed every 48 h to allow growth factors released by the cells to remain present. Tumouroids were always set as biological triplicates.

*Inhibitory Drug Treatment (UK-371,804).* UK-371,804 (PZ0344, Sigma Aldrich, Dorset, UK) was diluted to 10 μM in medium with 0.1 % DMSO vehicle. It was applied to the tumouroids every 48 h, starting on day 2.

*Generating a PLAU Knockout HCT116 Cell Line by CRISPR-Cas9.* Pre-designed crRNA, tracrRNA and Cas9 protein were ordered from IDT (Leuven, Belgium). Three RNA guides were tested, see Table 1 for guide sequences. Cas9-RNPs were transfected by nucleofection using Lonza’s 4D-Nucleofector^TM^ system. gRNAs were formed by hybridising crRNA and tracrRNA at 95 °C for 5 mins. RNP complexes were formed by mixing gRNA (61 μM) with Cas9 protein (61 μM, IDT), and incubating at room temperature for 20 min. In the meantime, the cells were detached and resuspended in 4D-Nucleofector^TM^ solution. The cells and RNP complexes were transferred to Nucleocuvettes^TM^ according to Lonza’s protocol. The cuvette strip was placed in the 4D-Nucleofector unit, and the SE protocol run. The cells were then transferred to a 6-well plate and incubated in a humidified incubator (37 °C, 5 % CO_2_) for 48 h, before screening for pooled guide editing efficiency. Monoclonal cell lines were generated by limiting dilution using the guide with the highest editing efficiency. Eight clonal populations were screened by SANGER sequencing and western blot to identify a knock-out. Table 2.

**Table 1:** Guides screened for the *PLAU* knockout. Designed by IDT.

crRNA Design ID	Sequence
Hs.Cas9.PLAU.1.AA	GCTTAACTCCAACACGCAAG
Hs.Cas9.PLAU.1.AB	CAGACAACCGGAGGCGACCC
Hs.Cas9.PLAU.1.AC	GGGAATGGTCACTTTTACCG

**Table 2:** Antibodies used for immunofluorescence staining.

Target	Reference	Dilution
Pan-Cytokeratin	Anti-Cytokeratin wide spectrum antibody Rb pAb (GeneTex, Irvine, California, USA)	1:200
Red 2° anti rabbit	DyLight® 594 IgG H&L ab96885 (Abcam, Cambridge, UK)	1:1000

*PCR, Sanger Sequencing, and ICE Analysis.* DNA was extracted from the edited cells using the DNeasy Blood & Tissue Kit (Qiagen, Hilden, Germany). PCR was run using primers found in Table S1, with Platinum™ II Hot-Start PCR Master Mix (2X) (Invitrogen, through Thermo Fisher Scientific, Loughborough). The PCR products’ size and purity were checked on an 1 % agarose gel, then purified using the QIAquick PCR Purification Kit (Qiagen, Hilden, Germany). The purified PCR products were sent for Sanger sequencing through Source Bioscience (Nottingham, UK). The sequences were analysed using Synthego’s ICE CRISPR Analysis Tool.

*Protein Extraction and Western Blot*. 2D cells were lysed for protein using RIPA buffer containing protease inhibitor cocktail at 1:100 dilution (both Sigma-Aldrich, Dorset, UK). Protein concentration was determined using the Pierce^TM^ BCA Protein Assay Kit (Thermo Fisher Scientific, Loughborough, UK). 30 μg of protein lysates in 4X fluorescent compatible sample buffer (Invitrogen, through Thermo Fisher Scientific, Loughborough) and 2 % β-mercaptoethanol (Sigma-Aldrich, Dorset, UK) were boiled at 99 °C for 5 min. Proteins were then separated on 4–12 % NuPAGE Bis-Tris polyacrylamide gels (Invitrogen, through Thermo Fisher Scientific, Loughborough), and transferred on to PVDF membranes (Sigma-Aldrich, Dorset, UK). Membranes were incubated with Superblock dry blend (TBS) blocking buffer (Thermo Fisher Scientific, Loughborough) for 1 h at room temperature on the shaker. The membranes were then incubated in primary antibody Human u-Plasminogen Activator (uPA)/Urokinase Antibody Goat IgG (AF1310, R&D Systems, Abingdon, UK) at 0.1 μg/mL at 4 °C overnight on a shaker, and in mouse beta-actin primary antibody (8H10D10, Cell Signalling, Danvers, USA) for 1 h at room temperature. After washes, the membranes were incubated with secondary antibodies at 1:10000 (IRDye® 680RD anti-Mouse IgG, #926–68070; IRDye® 800CW anti-Goat IgG, #926–32214, both from Licor Biosciences, Lincoln, USA) for 1 hr at room temperature. Membranes were imaged with the Li-COR CLx system (Licor Biosciences, Lincoln, USA).

*Atomic Force Microscopy.* AFM was performed on simple tumouroids, after 21 days of culture. Each experiment was set up with three biological replicates and acellular controls. On the day of AFM measurements, the cantilever used (RFESP-75, k ∼2 N/m, with a 50 μm of diameter glued glass bead, Bruker, Berlin, Germany) was calibrated in liquid, on glass, to determine sum and sensitivity. A CellHesion® 200 AFM (JPK BioAFM, Bruker Nano GmbH, Berlin, Germany) was used. The tumouroids were measured in liquid, and at room temperature (in Leibovitz's L-15, no phenol red, Gibco^TM^ through Thermo Fisher Scientific, Loughborough, UK). The cantilever used was a RFESP-75 (k ∼2 N/m, Bruker, Berlin, Germany) with a 50 μm of diameter glued glass bead (Cospheric LLC, California, USA). Each tumouroid was probed along a grid (4×4 map of 1500×1500 μm leading to a total of 16 measurements per sample). The set force was 700 nN, which insures a 10 to 15 μm indentation. Using the JPK BioAFM SPM data processing software, the Hertz model was fitted to the collected force curves to determine the Young’s Modulus E, assuming a Poisson ratio (ν) of 0.5. Stiffness measurements were normalised to the acellular controls.

*RNA Extraction and Quantitative Polymerase Chain Reaction (qPCR).* RNA was extracted from the cells grown in 3D tumouroids. The entire gel was placed in trizol to extract RNA (phase separation TRI Reagent® and chloroform method [37]). Then, cDNA was transcribed using the High-Capacity cDNA Reverse Transcription Kit (Applied Biosystems^TM^ through, Fisher Scientific, Loughborough, UK). Primers used for qPCR can be found listed in Table S2. qPCR was performed using iTaq^TM^ Universal SYBR® Green Supermix (Applied Biosystems^TM^ through, Fisher Scientific, Loughborough, UK). Per biological replicate, three technical replicates were performed and averaged. Relative gene expression was calculated using the ΔCt method, normalising to house-keeping gene GAPDH [38].

*Enzyme-Linked Immunosorbent Assay (ELISA).* Media samples were collected in triplicates for each condition and stored at −80 °C. The R&D Systems (Abingdon, UK) Human uPA Quantikine ELISA Kit (DUPA00) was used according to the manufacturer’s instructions. Per biological replicate, technical duplicates were performed and averaged. Results were read using the Tecan Infinite® M Plex plate reader (Männedorf, Switzerland).

*PrestoBlue*® *Metabolic Activity Assay*. At time point of interest, 10 % PrestoBlue® (Invitrogen^TM^, through Thermo Fisher Scientific, Loughborough) in medium was added to the each well. The plate was then incubated for 2 h at 37 °C. The solution from each well was transferred to a black plate in duplicates and fluorescence was measured by the Tecan Infinite® M Plex plate reader (Männedorf, Switzerland).

*CellTiter Glo Metabolic Activity Assay*. CellTiter-Glo® 3D Viability-Assay (Promega, Southampton, UK) is a metabolic activity assay used for 3D samples of over 7 days, as it penetrates within a spheroid better than PrestoBlue®. At time point of interest, CellTiter-Glo® was mixed (1:1) with the media in each well. The plate was shaken for 5 min and then incubated on the benchtop for 25 min. The solution was transferred to a white plate in duplicates and luminescence was measured by the Tecan Infinite® M Plex plate reader (Männedorf, Switzerland).

*uPA activity assay.* uPA Activity Assay Kit ECM600 (Sigma-Aldrich, Dorset, UK) was used to determine uPA inhibitor efficacy. HCT 116 conditioned media was applied to the assay, in biological replicates and technical duplicates. Three conditions were tested and compared: 1. No drug, 2. 1 μM drug and 3. 5 μM drug (all with 0.1 % DMSO vehicle). After 24 h, uPA activity was determine via a plate reader (Tecan Infinite® M Plex plate reader, Männedorf, Switzerland).

*Immunofluorescent Staining and Imaging.* At day 21, tumouroids were fixed with 10 % neutrally buffered formalin (Genta Medical, York, UK) for 30 min and then washed and stored in PBS (Gibco^TM^, through Fisher Scientific, Loughborough, UK). Before staining, all samples were blocked for 1 h using 0.2 % triton X-100 and 1 % bovine serum albumin (BSA) (both from Sigma-Aldrich, Dorset, UK) in PBS. Then, the primary antibody, diluted in blocking solution (see table X for dilutions), was applied to the samples. They were incubated overnight at 4 °C. The secondary antibody incubation was carried out the next day for 2.5 h, at room temperature. A DAPI counterstain was applied 20 min before imaging (NucBlue^TM^, (Invitrogen^TM^ through Fisher Scientific, Loughborough, UK). Samples were imaged on the Zeiss AxioObserver using Zeiss ZEN software (Zeiss, Oberkochen, Germany).

*Invasion Quantification.* All complex tumouroids were imaged on the Zeiss AxioObserver and software (Zeiss, Oberkochen, Germany), in brightfield mode. In order to measure the outgrowth, i.e the invasion, from the initial ACM-stroma border into the stroma, four images were taken at a 2.5x magnification at randomised locations. Overall area of invasion and maximum distance of invasion was calculated for each image, using FIJI ImageJ software.

*Statistical Analysis.* All data was analysed and visualised using GraphPad Prism 9 software. All AFM data was non-parametric, therefore Mann-Whitney (2 groups) or Kruskal-Wallis (three groups or more) tests were used. All other data was first tested for normality using the Shapiro-Wilk test. If normally distributed and parametric, a *t*-test was used (2 groups) or a one-way ANOVA test with Dunnet’s post hoc (for multiple groups). All n numbers, p-values and tests conducted are mentioned in figure caption. Data in graphs is shown as mean with standard error mean (SEM) for all biological testing and as mean with standard deviation (STDEV) for mechanical testing. P-value significance is indicated as: 0.05> *, 0.01> **, 0.001> *** and 0.0001> ****.

## Declaration of Competing Interest

The authors declare that they have no known competing financial interests or personal relationships that could have appeared to influence the work reported in this paper.

## Data Availability

Data will be made available on request.
