# Supplementary material for: Urokinase-type plasminogen activator (uPA) regulates invasion and matrix remodelling in colorectal cancer
Source: Matrix Biol Plus. 2023 Nov 15;19-20:100137. doi: 10.1016/j.mbplus.2023.100137 (PMC10667746; doi:10.1016/j.mbplus.2023.100137)
Supplement: Supplementary data 1 [file mmc1.docx]

***Supplementary information***

**Urokinase-type plasminogen activator (uPA) regulates invasion and matrix remodelling in colorectal cancer.**

Auxtine Micalet^1,2^, Luke J. Tappouni^3^, Katarzyna Peszko^1^, Despoina Karagianni^4^, Ashley Lam^1^, John R. Counsell^3^, Sergio A. Quezada^4^, Emad Moeendarbary^2,5*^, Umber Cheema^1*^

^1^ UCL Centre for 3D Models of Health and Disease, Department of Targeted Intervention, Division of Surgery and Interventional Science, University College London, Charles Bell House, 43-45 Foley Street, W1W 7TS, London, United Kingdom.

^2^ Department of Mechanical Engineering, University College London, Gower Street, WC1E 6BT, London, United Kingdom.

^3^ UCL Centre for Targeted Cancer Therapies, Department of Targeted Intervention, Division of Surgery and Interventional Science, University College London, Charles Bell House, 43-45 Foley Street, W1W 7TS, London, United Kingdom.

^4^ Immune Regulation and Tumour Immunotherapy Group, UCL Cancer Institute, University College London, 72 Huntley Street, WC1E 6DD, London, United Kingdom.

^5^ 199 Biotechnologies Ltd, Gloucester Road, London, W2 6LD UK.

* These authors share senior authorship

Corresponding author:

Umber Cheema: [u.cheema@ucl.ac.uk](mailto:u.cheema@ucl.ac.uk)


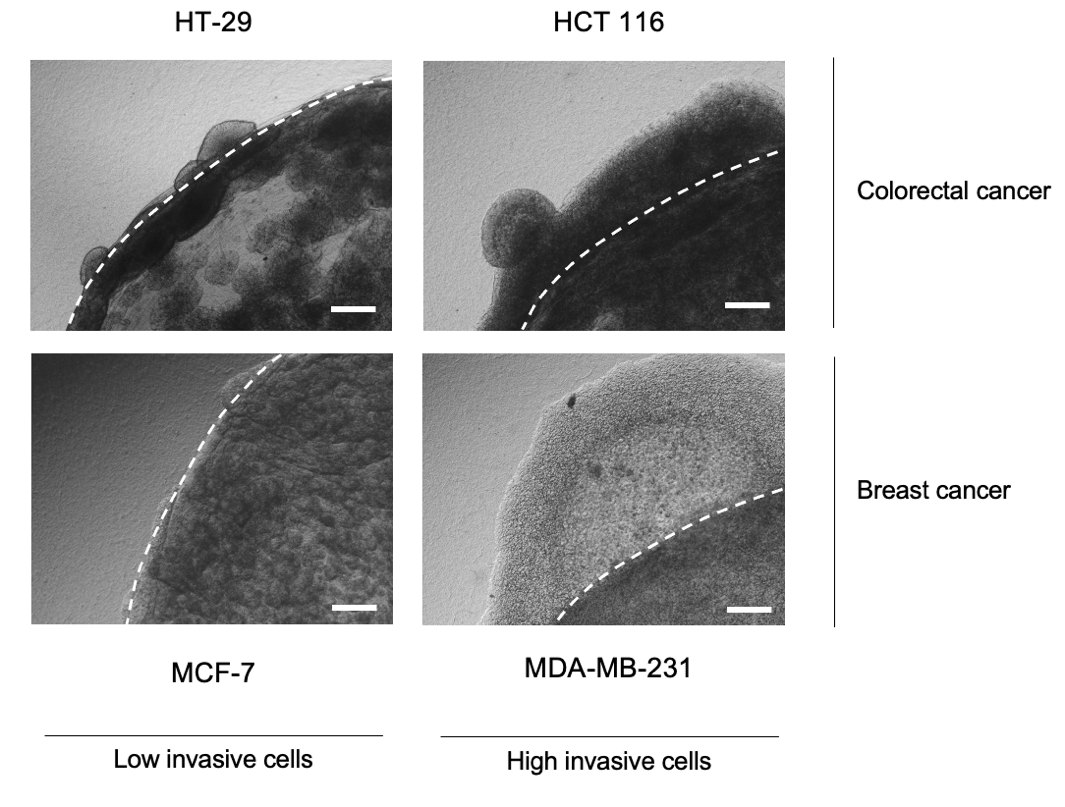


**Figure S1 – Invasion patterns of HT-29, HCT 116, MCF-7 and MDA-MB-231 cells into an acellular stroma after 21 days.** Showing that HT-29 and MCF-7 are less invasive than their counter-parts, HCT 116 and MDA-MB-231. The ACM edge is drawn as a dashed line. Scale bar = 200 µm.

**
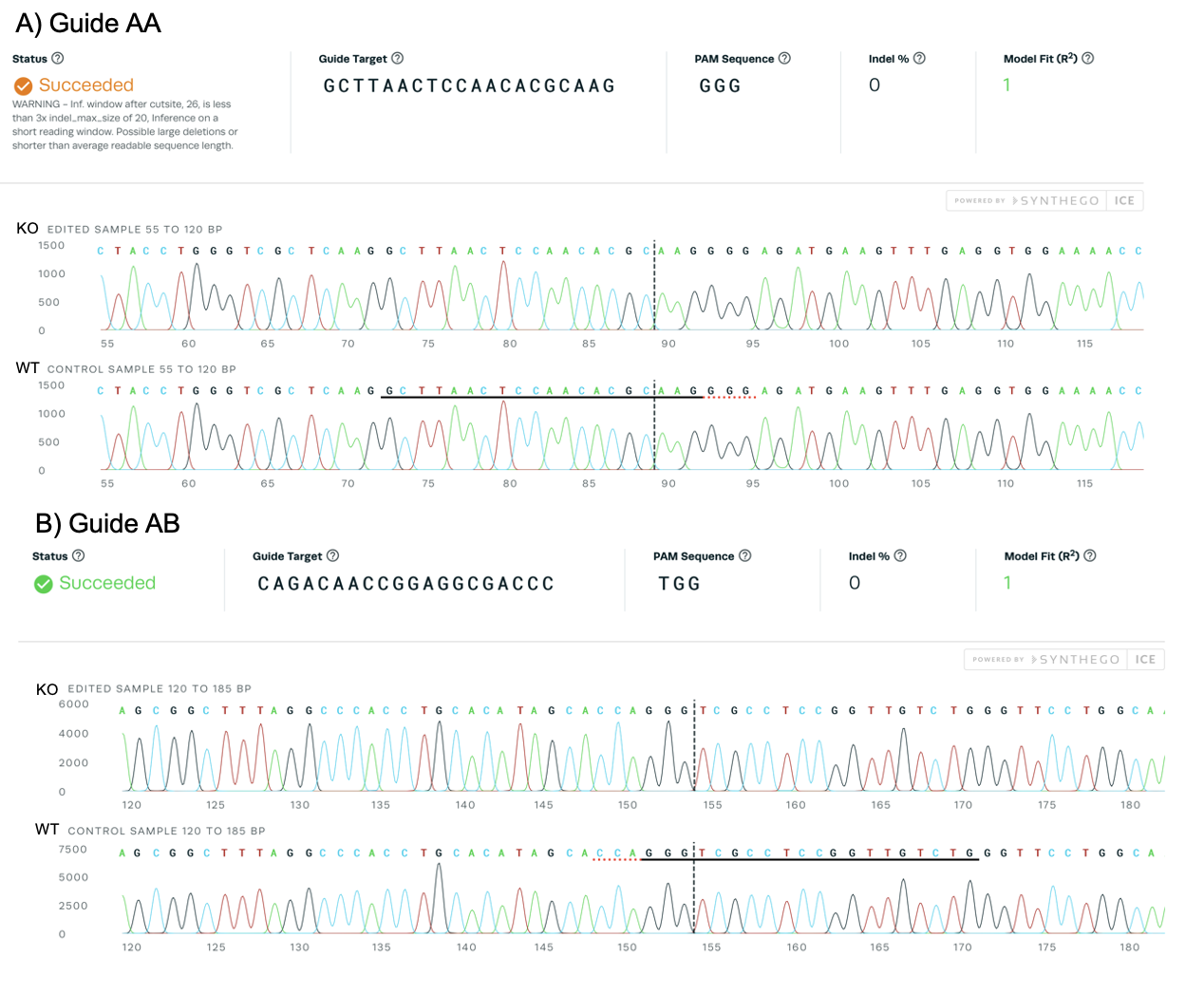
**

**Figure S2 – Knockout efficiency of the other two RNA guides tested (AA and AB).** ICE analysis demonstrating the efficiency of **A)** RNA guide AA and **B)** RNA guide AB. Neither guides created a gene knock-out. The sequencing traces show no indel, the indel % score is zero. The knock-out score is therefore zero. The horizontal black underlined region represents the guide sequence, preceded by the PAM sequence underlined with a red dotted line. The vertical black dotted line represents the cut site.

**
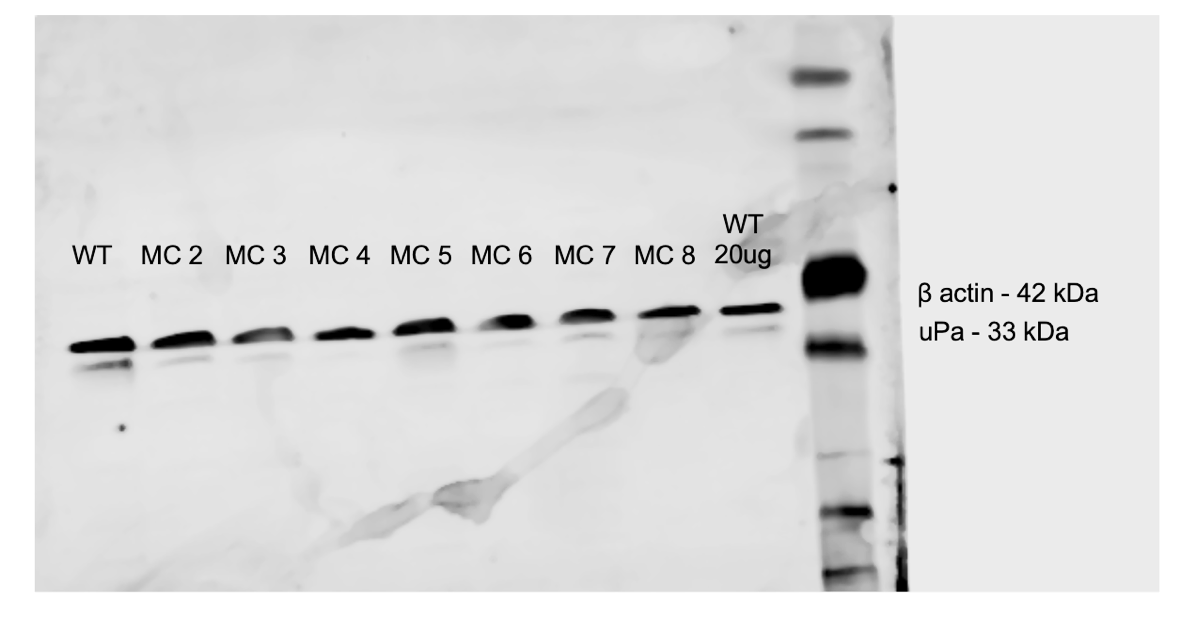
**

**Figure S3 – uPA expression in each monoclonal (MC) populations.** Western Blot of the uPA expression in each MC population. Housekeeping protein is Beta actin. From left to right: WT HCT 116, MC 2, MC 3, MC 4, MC 5, MC 6, MC 7, MC 8 and WT at half the protein concentration (20 µg, instead of 40 µg for the other wells). Only clonal population 4 shows no uPA presence.


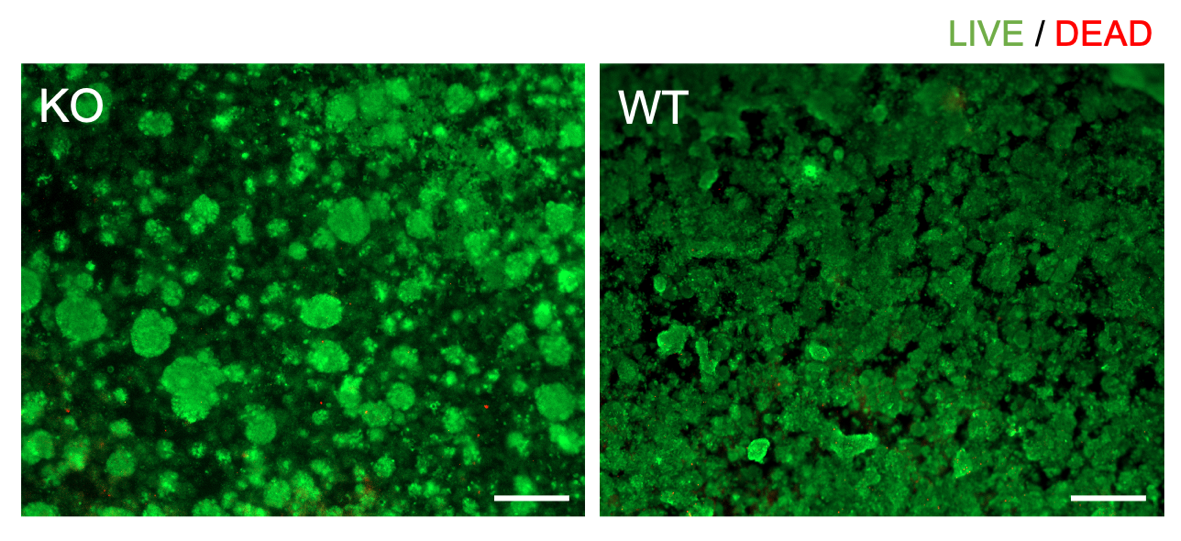


**Figure S4 – Live/dead staining of tumouroids at day 21.** **Left:** Tumouroids made with *PLAU* KO HCT 116 cells. **Right:** Tumouroids made with WT HCT 116 cells. Green = live cells; red = dead cells. Scale bar = 200 µm.


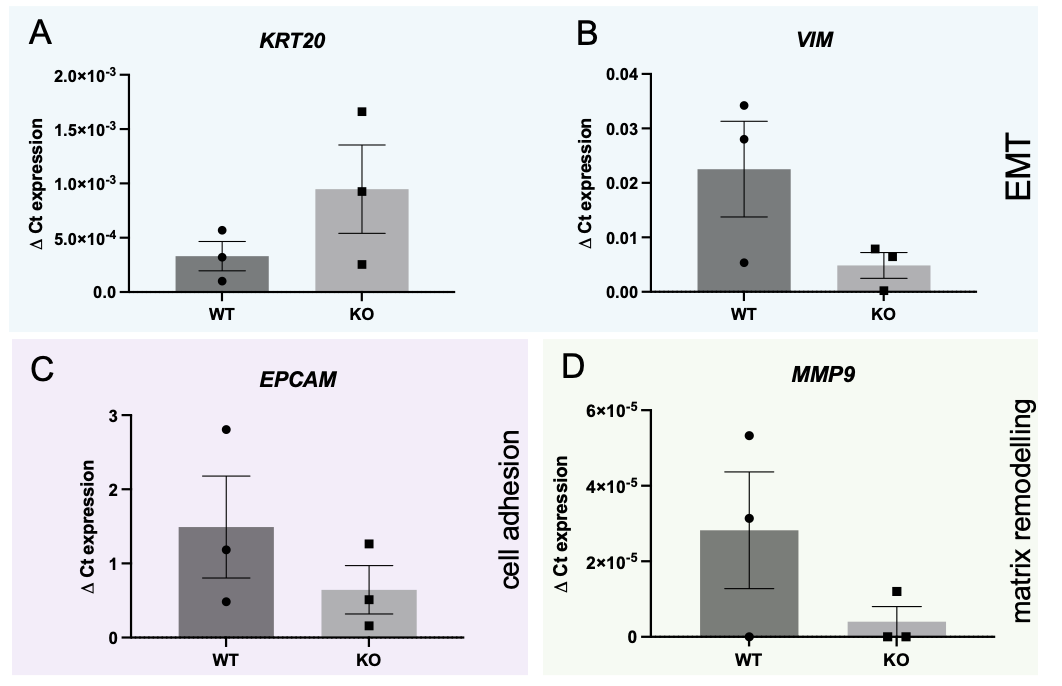


**Figure S5 – Gene expression of invasive markers in *PLAU* knockout HCT 116 cancer cells.** Relative gene expression (ΔCt, normalised to *GAPDH*) of cancer invasion markers at day 14 in the tumouroid for HCT 116 WT and HCT 116 *PLAU* KO cells. **A)** Cytokeratin 20 (*KRT20),* an epithelial marker **B)** Vimentin (*VIM*), a mesenchymal marker **C)** *EPCAM*, a cell-cell adhesion marker **D)** *MMP9*, an ECM degradation marker. Significance tested using an un-paired t-test for A,B,C and Mann-Whitney test for D.

**Figure S6 – Cell viability at increasing UK-37,804 uPA inhibitor concentration.** 2D Presto Blue assay of HCT 116 and HT-29 cells, checking for cell viability for UK-37,804 concentrations from 0 to 100 µM (all with 0.1% DMSO as a vehicle). Cells were treated twice, once on day 2 and once again on day 4 before the assay was performed.


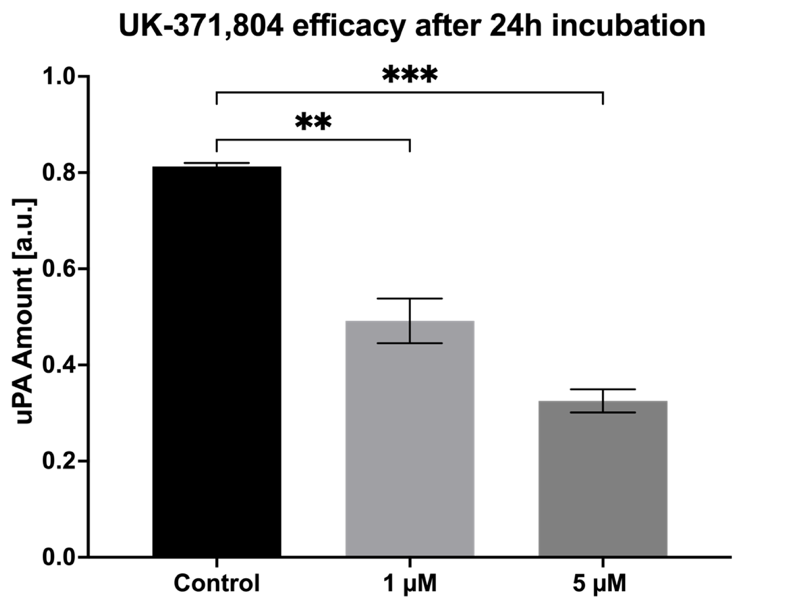


**Figure S6 – UK-37,804 efficacy.** 2D uPA activity assay. HCT 116 conditioned media was applied to the assay 1. without drug, 2. With 1 µM drug and 3. With 5 µM drug (all with 0.1% DMSO vehicle). After 24 hours, uPA activity was determine via a plate reader. At 1 µM we observed a 39% decrease in protein activity, and a decrease of 60% for 5 µM. Showing Kruskal-Wallis significance, All p-value significance is indicated as: ** <0.01, *** <0.001.

| Primer pair | Sequence F’ (5’-3’) | Sequence R’ (5’-3’) |
| --- | --- | --- |
| For AB & AC | CTGGCAGGTCTGAAACGACA | GTCCTCCTGTGATGGAATAAGG |
| For AA | AAGGAAGAAGTGGCAGATTTCA | CAGCGCTGTAGTCCTTGTGTAG |

**Table S1 – Primers used to amplify around the expected cut-sites (for guides AA, AB and AC)**

**Table S2 – qPCR primers and their sequences**.

|  | Sequence F’ (5’-3’) | Sequence R’ (5’-3’) |
| --- | --- | --- |
| *KRT20* (IJMS Pape 2023^36^) | ATTGCTACTTACCGCCGCCTTC | GACACGACCTTGCCATCCACTAC |
| *VIM* (AHM Micalet 2022^5^) | TCTCTGGCACGTCTTGACCTTG | CGATTTGGACATGCTGTTCCTG |
| *SNAI1* | AGCCGTGCCTTCGCTGAC | CGGACTCTTGGTGCTTGTGGAG |
| *EPCAM* (EJC Pape 2019^37^) | TTGCTGTTATTGTGGTTGTGGTG | CCCATCTCCTTTATCTCAGCCTTC |
| *CDH1* (IJMS Pape 2023^36^) | GAAGAAGGAGGCGGAGAAGAGG | CATGAGGGTTGGTGCAACGTC |
| *RHOA* | GTGATGGAGCCTGTGGAAAGAC | ATCGGTATCTGGGTAGGAGAGG |
| *HPSE* (EJC Pape 2019^37^) | ﻿TAAGACCTTTGGGACCTCATGG | ﻿CAGATGCAAGCAGCAACTTTGG |
| *MMP9* (AHM Micalet 2022^5^) | CAGTCCACCCTTGTGCTCTTCC | TTCGACTCTCCACGCATCTCTG |
| *GAPDH* (Al Hosni iScience 2022^38^) | GCTCTCTGCTCCTCCTGTTC | CGACCAAATCCGTTGACT CC |
